# Enhancement of *Apostichopus japonicus* peptide flavor through bacterial and enzyme co-fermentation (BECF) and the identification of novel antioxidant peptides in the fermented product

**DOI:** 10.1016/j.fochx.2025.102323

**Published:** 2025-02-26

**Authors:** Zhiqiang Shu, Gongming Wang, Yuexin Jing, Chunna Jiao, Leilei Sun, Hui Huang, Yue Li, Jian Zhang

**Affiliations:** a Shandong Marine Resource and Environment Research Institute, Yantai 264006, PR China; b Department of Food Science and Technology, Shanghai Ocean University, Shanghai 201306, PR China; c Yantai Key Laboratory of Quality and Safety Control and Deep Processing of Marine Food, Yantai 264006, PR China; d Yantai Key Laboratory of Characteristic Agricultural Bioresource Conservation & Germplasm Innovative Utilization, School of Life Sciences, Yantai University, Yantai 264005, PR China

**Keywords:** *Apostichopus japonicus*, Fermentation, Enzymolysis, Process optimization, Gas chromatography-ion migration spectroscopy, Antioxidant peptide, 2,2-Diphenyl-1-picrylhydrazyl, 2,2′-azino-bis-(3-ethylbenzothiazoline-6-sulfonic acid), Anhydrous ethanol, Trichloroacetic acid, Acetonitrile, Carboxylic acid, Vitamin C, Potassium persulfate, Hydrogen peroxide

## Abstract

In this study, we optimized the BECF process parameters by single-factor experiments and response surface methodology (RSM). Additionally, various analytical techniques were employed to determine the volatile flavor compounds, amino acid composition, and peptide sequences of the fermented product. The antioxidant activities of 10 peptides were evaluated via free radical scavenging assays. The results indicated that the optimal BECF conditions for *Apostichopus japonicus* body wall (AJBW) were as follows: 2.3 % bacterial inoculum, fermentation for 31 h at 30 °C, 463 U/g enzyme dosage, and enzymatic hydrolysis at 50 °C for 4 h. Gas chromatography-ion mobility spectrometry analysis revealed a significant reduction in aldehydes, which impart a pungent odor, in the co-fermented product (AJM) as compared to the control. While the content of alcohols, ketones, and esters, which contribute to aromatic flavors, was significantly increased. The content of essential amino acids in AJM, as analyzed through an automatic amino acid analyzer, was slightly higher compared to that in AJBW. Liquid chromatography-tandem mass spectrometry identified a total of 808 sea cucumber peptide fragments with high confidence. DPPH, ABTS, and hydroxyl radical scavenging assays revealed that peptides LFW and LFPW exhibited the strongest antioxidant activities. Molecular docking studies showed significant hydrogen-bonding interactions. In conclusion, BECF is an effective strategy for enhancing the flavor of *A. japonicus* peptide.

## Highlights

• First significant improvement of *Apostichopus japonicus* peptide flavor by bacterial enzyme co-fermentation (BECF).
• GC-IMS-based reveals the mechanism of improving the flavor of sea cucumber peptides by BECF technology.
• Identified 808 novel peptides with 10 showing potent antioxidant activity via LC-MS/MS and PeptideRanker.
• Peptides LFW and LFPW demonstrated the highest antioxidant activities, confirmed by DPPH, ABTS, and hydroxyl radical scavenging assays.

## 1 Introduction

Sea cucumber is a valuable marine product with both nutritional and medicinal benefits. Its body wall contains various bioactive substances, including sea cucumber polysaccharides, saponins, peptides, and phenolic compounds (Zhao et al., 2018). Among these, sea cucumber peptides have garnered significant attention due to their high content, low molecular weight, and ease of digestion and absorption. Sea cucumber peptides exhibit various biological activities, including antioxidant (Guo et al., 2020), antibacterial (Thawabteh et al., 2023), lipid-lowering (Xu et al., 2018), antihypertensive (Liang et al., 2022), and immune-boosting effects (He et al., 2016), making them a focal point in marine bioactive substance research. The preparation of sea cucumber peptides primarily involves enzymatic hydrolysis and fermentation methods. The enzymatic hydrolysis method employs various proteases to shorten peptide chains and enhance the hydrolysis of sea cucumber proteins. Its potent hydrolytic capability efficiently releases sea cucumber peptides. However, enzymatic hydrolysis can also produce volatile compounds with fishy or off-flavors, such as trimethylamine and sulfides, which can affect the product's taste and consumer acceptance (Zhang et al., 2020). Fermentation involves hydrolyzing proteins using various enzymes and acids produced by fermenting bacteria. This process converts substrate nutrients into volatile flavor compounds and amino acids, such as esters and alcohols, mainly through microbial metabolic pathways. Although it can enhance product flavor, its disadvantages include being time-consuming and having low hydrolysis efficiency (Chai et al., 2020). To overcome the limitations of single methods, bacterial and enzyme co-fermentation technology has emerged. This technology combines the advantages of enzymatic hydrolysis and fermentation through careful design, significantly improving flavor and increasing the peptide content of sea cucumbers. As a result, research on BECF technology has been increasing annually in fields such as feed, food, and herbal medicine, demonstrating its broad application prospects (Ran, et al., 2023; Yu et al., 2024). However, research on the BECF of sea cucumber body wall and the subsequent analysis of its products remains limited, necessitating further in-depth investigations.

Analyzing the changes in volatile flavor compounds in seafood subjected to enzymatic hydrolysis or fermentation using gas chromatography-ion mobility spectrometry (GC-IMS) is a prevalent research method (Li et al., 2023). Additionally, liquid chromatography-tandem mass spectrometry (LC-MS/MS) can rapidly identify and analyze amino acid sequences of peptides at trace levels, facilitating the detection of numerous oligopeptides (Li, Lu, et al., 2022). Building upon preliminary laboratory studies (Zou et al., 2023), this research employs salted *Apostichopus japonicus* as raw material, and *Bacillus amyloliquefaciens* and a complex protease was employed for the synergistic fermentation of its body wall. The fermentation process parameters were optimized using single-factor experiments and RSM with the composite scores of sensory score, peptide content and free amino acid content as evaluation indexes. Furthermore, changes in volatile flavor compounds pre- and post- fermentation were analyzed using GC-IMS; amino acid changes were determined using an automatic amino acid analyzer; and peptide sequences in the fermentation product AJM were identified using LC-MS/MS. Following bioinformatics analysis, 10 peptides with potential biological activities were identified. And its antioxidant activities were evaluated through DPPH, ABTS, and hydroxyl radical scavenging assays. In this study, RSM was integrated with the BECF process for the first time to achieve dual enhancement of both flavor and functionality in sea cucumber peptide products. This approach offers a novel strategy for the development of sea cucumber peptide-based functional foods with reduced fishy taste and enhanced bioactivity. Furthermore, this study not only provides an effective method for the development of high-quality sea cucumber products but also contributes theoretical support for the advancement and application of functional foods.

## 2 Materials and methods

### 2.1 Materials and chemicals

The salted *Apostichopus japonicus* used in this study were purchased from Yantai Wanshiruyi Foods Co., Ltd. (Yantai, China). *Bacillus amyloliquefaciens* (strain no. 10035) was sourced from the China Industrial Strain Preservation Center (Beijing, China). Complex protease (1.6 AU-N/g) was supplied by Novozymes (Copenhagen, Denmark). The Lowry method protein concentration assay kit, along with the DPPH, ABTS, and hydroxyl radical scavenging activity assay kits, were purchased from Beijing Solarbio Science & Technology Co., Ltd. (Beijing, China). The total amino acid assay kit was obtained from Nanjing Jiancheng Bioengineering Institute (Nanjing, China). LB liquid medium, methanol, formic acid, acetonitrile, anhydrous ethanol, 30 % hydrogen peroxide (H₂O₂), and ascorbic acid were purchased from Sinopharm Chemical Reagent Co., Ltd. (Beijing, China). All chemicals and solvents used in this study were of analytical grade.

### 2.2 Sample pretreatment

Rinse the salted *Apostichopus japonicus* thoroughly and soak it in deionized water to remove the salt. Remove the sand mouth, wash it, and cut the sea cucumber into pieces. Mix it with an equal amount of deionized water and homogenize it using a 150-mesh colloid mill at 3000 r/min for 5 min. This mixture serves as the untreated homogenate sample, referred to as AJBW, and stored at 4 °C.

The protein content of AJBW homogenate was determined by the Lowry method using bovine serum albumin as a standard. The protein content was found to be 4.54 ± 0.24 g per 100 g of homogenate (wet weight).

### 2.3 Single-factor fermentation experiment

Inoculate the frozen *Bacillus amyloliquefaciens* into LB liquid medium and incubate at 37 °C for 18 h. After two generations of activation, use the culture as seed culture and store at 4 °*C. prior* to each use, reactivate the seed culture for one generation until it reaches an OD_600_ of 0.4. To prevent interference from other microorganisms during the fermentation process, the raw materials were autoclaved before using. After that, 50 g of homogenized sample was weighed and sterile water was added in the ratio of 1:3, adjusting the pH to 7.2. *Bacillus amyloliquefaciens* was subsequently inoculated, and a composite score incorporating sensory evaluation, polypeptide content, and total free amino acid content was used as the evaluation index to assess the effects of varying inoculum volume (bacterial suspension to sample volume ratio), fermentation temperature, and fermentation time on the composite score of the fermentation products. Based on a review of relevant literature and preliminary experiments (Wang et al., 2024), the inoculum volumes of *Bacillus amyloliquefaciens* were set at 0.5, 1, 3, 5, and 7 %, with fermentation temperatures of 20, 25, 30, 35, and 40 °C, and fermentation times of 12, 24, 36, 48, and 60 h. The activity of the inoculated *Bacillus amyloliquefaciens* during fermentation was monitored by measuring the optical density (OD_600_) at regular intervals, and the bacterial colony count was determined using the plate-count method. To verify the identity of the fermentation microbiota, 16S rRNA gene sequencing was performed on samples taken at various time points during the fermentation process to ensure the presence and stability of the inoculated *Bacillus amyloliquefaciens*.

### 2.4 Single-factor enzyme hydrolysis experiment

Prepare a specific volume of fermentation broth according to the procedure outlined in Section 2.3. Then, weigh 50 g of the fermentation broth and add an appropriate amount of complex protease. The mixture undergoes enzymatic hydrolysis at pH 6 and an suitable temperature for a defined period. The enzyme is inactivated by heating in a boiling water bath for 10 min. After centrifugation at 7000 r/min for 10 min, the supernatant is collected and referred to as AJM. The effects of different enzyme dosage, enzymatic hydrolysis time, and enzymatic hydrolysis temperature on the AJM composite score were investigated, using the composite scores of sensory evaluation, polypeptide content, and total free amino acid content as evaluation indices. By searching related literature and conducting pre-experiments (Sun et al., 2022), the addition amounts of complex protease were set at 250, 500, 750, 1000 and 2000 U/g (calculated based on homogenate mass and enzyme activity), the enzymatic hydrolysis times was set at 2, 3, 4, 5 and 6 h, and the enzymatic hydrolysis temperatures were set at 30, 40, 50, 60 and 70 °C.

The solid residues generated during centrifugation were collected and subjected to composting for biodegradation. Liquid wastes, including fermentation broth and enzyme solutions, were neutralized to pH 7.0 and treated via aerobic digestion prior to disposal, in compliance with local environmental regulations. AJBW was defined as unfermented sea cucumber body wall homogenate, whereas AJM was the product of fermentation by *Bacillus amyloliquefaciens* (2.3 % inoculum) for 31 h (30 °C), followed by enzymatic hydrolysis by a complex protease (463 U/g) for 4 h at 50 °C.

### 2.5 Response surface methodology

Building on the single-factor fermentation and enzymatic hydrolysis experiments, Design-Expert software was employed to extend the investigation using the Box-Behnken center composite design principles. This study investigates the effects of enzyme dosage (A), bacterial inoculum volume (B), and fermentation time (C) on the degradation of the body wall of *Apostichopus japonicus*. The weighted composite score was used as the response variable in a three-factor, three-level response surface experiment designed to identify the optimal conditions for the combined fermentation and enzymatic degradation of the body wall of *Apostichopus japonicus*. The levels of the factors for response surface optimization are detailed in Table 1. In addition, the ranges of bacterial inoculum, fermentation time and enzyme dosage were set based on literature reports (Wang et al., 2024; Zou et al., 2023) and results of the single-factor pre-experiment.

**Table 1:** Independent variables and coded values in Box-Behnken Design for single-factor experiments.

Independent variables	Symbol	Levels		
		−1	0	1
enzyme dosage (U/g)	A	250	500	750
bacterial inoculum volume (%)	B	1	3	5
fermentation time (h)	C	24	36	48

### 2.6 Sensory evaluation

The sensory evaluation standard was referred to the method of Wang tao et al. with slight modifications based on the parameters (Wang et al., 2020). Ten trained food researchers, representing various age groups and possessing normal taste and olfaction, were selected to conduct sensory evaluation. These researchers assessed and scored the samples based on three attributes: fishy odor, aroma, and taste, as detailed in Table 2. Given the varying impacts of these three attributes on the quality of the degradation solution, weight coefficients of 3, 3, and 4 were assigned to fishy odor, aroma, and taste, respectively. The sample score was calculated using the formula: $\mathrm{Sample Score}=\left(\mathrm{Fishy Odor Score}\times 3\right)+\left(\mathrm{Aroma Score}\times 3\right)+\left(\mathrm{Taste Score}\times 4\right)$, with a maximum possible score of 100 points. Each sample was tested in triplicate, with the highest and lowest scores excluded. The average of the remaining scores was calculated and used as the final sensory rating for each sample. This study was conducted in accordance with the ethical guidelines of Shanghai Ocean University. All participants provided informed consent prior to their participation in the sensory evaluation, and their participation was voluntary. The study was designed to ensure the confidentiality and privacy of all participants. No ethical violations occurred during the course of the study.

**Table 2:** Sensory evaluation criteria.

Items	scoring criteria			
	1–2 points	3–5 points	6–8 points	9–10 points
Fishy Odor (30 points)	Strong fishy odor	Moderate fishy odor	Mild fishy odor	Nearly absent fishy odor
Aroma (30 points)	Excessive fermentation flavor and acidity, accompanied by a noticeable off-flavor	Possesses the characteristic odor of sea cucumber without any undesirable odors	Exhibits the characteristic odor of sea cucumber with a fresh, aromatic scent produced during fermentation	Features the characteristic odor of sea cucumber, with a pleasant fruity aroma and a refreshing acidic taste
Taste (40 points)	Poor taste, excessively bitter and salty, with an off-flavor	Moderate taste, slightly bitter and salty	Suitable taste, mildly sweet and slightly salty	Smooth taste, with a balanced sweet and sour profile and a pleasantly aromatic flavor

### 2.7 Determination of peptide content

The peptide content was quantified following the method outlined by Lu Wei et al., with appropriate modifications (Lu et al., 2005). Initially, 1 mL of the degradation solution was mixed with 1 mL of 10 % (*w*/*v*) trichloroacetic acid solution. The mixture was incubated at 4 °C for 10 min, followed by centrifugation at 10,000 r/min for 10 min to obtain the supernatant. Subsequently, the sample's absorbance was measured at 650 nm according to the Lowry method using a protein concentration determination kit. The peptide concentration was then calculated using the standard curve (y = 0.8312× - 0.0039, R^2^ = 0.991).

### 2.8 Determination of total free amino acid content

The total concentration of free amino acids was determined using the total amino acid assay kit, following the manufacturer's instructions.

### 2.9 Composite score calculation

Composite Score Calculation: A multi-criteria weighted scoring method was utilized, with the composite score serving as the evaluation metric. The weighting coefficients for sensory evaluation, polypeptide content, and total free amino acid content were set at 0.50, 0.25, and 0.25, respectively. The composite score was calculated using the following formula:

$$\text{Composite Score}=\left(\text{Sensory Evaluation}\times 0.50\right)+\left(\text{Polypeptide Content}\times 0.25\right)+\left(\text{Total Free Amino Acid Content}\times 0.25\right)$$

To eliminate dimensional differences, we standardized the peptide content and free amino acid content using the maximum value method, converting them into dimensionless relative scores before performing weighted summation. The formulas for standardized treatment of peptide content and free amino acid content was as follows:

$$\mathrm{Standardized value}=\frac{\mathrm{Measured value}}{Ma\mathrm{ximum value within the group}}\times 100$$

### 2.10 Determination of volatile flavor compounds

Volatile flavor compounds were determined using the GC-IMS method, with minor modifications based on the method (Li, Dong, et al., 2022). Precisely weigh 2.0 g of the sample and place it into a 20 mL headspace vial. Seal the vial and incubate it in a shaker at 50 °C and 500 rpm for 15 min. The headspace sampling port temperature was set to 55 °C, with an injection volume of 0.5 mL, using high-purity nitrogen (N₂) as the carrier gas. A WAX column (RESTEK, USA) was used for gas chromatography, with the column temperature set at 60 °C and a total run time of 30 min. The ion mobility spectrometry detection temperature was set to 45 °C, with high-purity N₂ as the drift gas at a flow rate of 150 mL/min.

### 2.11 Determination of amino acid composition and content

The amino acid composition and content was determined according to the method specified in GB 5009.124–2016, “Determination of Amino Acids in Food,” using an automated amino acid analyzer (Hitachi L-8900, Japan) (Lu et al., 2022). The performance metrics, including linearity, recovery rate, and relative standard deviation, complied with the stipulated criteria.

### 2.12 Identification of sea cucumber peptide sequences

AJM was separated according to the method described by Zhang et al. (Zhang et al., 2017). A Minimate TM tangential flow ultrafiltration system (Pall Corporation，Port Washington，NY，USA) equipped with a 5 kDa cutoff membrane was used to collect fractions with molecular masses below 5 kDa. Subsequently, a dialysis bag with a 100 Da cutoff and a diameter of 25 mm was employed to eliminate low-molecular-weight impurities, resulting in purified sea cucumber peptides.

The amino acid sequence of AJM was analyzed using LC-MS/MS. A self-packed column with an inner diameter of 150 μm and a length of 150 mm, packed with Acclaim PepMap RPLC C18 (1.9 μm particle size, 100 Å pore size) (Thermo Scientific, USA), was used for liquid chromatography analysis. Mobile phase A consisted of 0.1 % formic acid in water, while mobile phase B was composed of 80 % acetonitrile with 0.1 % formic acid. The flow rate was maintained at 600 nL/min, and the total runtime for a single separation was 66 min. The gradient elution program was as follows: the initial proportion of mobile phase B was 4 %, increased to 8 % at 2 min, 28 % at 45 min, and 40 % at 55 min; it then rapidly increased to 95 % at 56 min and was maintained until 66 min. Mass spectrometry analysis was conducted using an electrospray ionization combined ion trap Orbitrap mass spectrometer (Q-Exactive，Thermo Scientific, USA), with the spray voltage set to 2.2 kV, the capillary temperature at 270 °C, and the scan range from 100 to 1500 *m*/*z*. The acquired raw mass spectrometry data were processed using Byonic software, with the target protein database searched based on the sample type.

### 2.13 Peptide sequence analysis and activity prediction

The bioactivity of the identified peptides was predicted using PeptideRanker (http://distilldeep.ucd.ie/PeptideRanker/), with scores approaching 1 indicating a greater likelihood of bioactivity (Fan et al., 2022). Peptide segments with PeptideRanker scores exceeding 0.95 were selected for further analysis. BIOPEP-UWM (https://biochemia.uwm.edu.pl/biopep-uwm/), encompassing all reported bioactive peptides, was utilized to ascertain the novelty of the obtained peptides. The physicochemical properties of the peptides were predicted using ProParam (http://web.expasy.org/protparam/). ToxinPred (http://crdd.osdd.net/raghava/toxinpred/) was employed to predict peptide toxicity based on Swiss-Prot data. AlgPred (http://www.imtech.res.in/raghava/algpred/) was utilized to evaluate the allergenicity of the peptides based on their amino acid sequences.

### 2.14 Evaluation of antioxidant activity in vitro

The antioxidant activity of the screened peptides was evaluated using DPPH, ABTS, and hydroxyl radical scavenging assays. The ability to scavenge DPPH free radicals was determined according to the method described by Mirzapour-Kouhdasht et al., with slight modifications (Mirzapour-Kouhdasht et al., 2022). A total of 100 μL of peptide solution was mixed with 100 μL of 0.1 mmol/L DPPH-ethanol solution and incubated at 37 °C for 30 min in the dark. The absorbance was measured at 517 nm. The negative and positive controls were deionized water and Vc, respectively. The DPPH scavenging rate was calculated using the following formula:

$$\mathrm{DPPH}+\mathrm{scavenging rate}\%=1-\frac{A_{2}}{A_{1}}\times 100\%$$

where A_1_ is the absorbance of the negative control and A_2_ is the absorbance of the peptide sample.

The ABTS free radical scavenging capacity was determined using the method of Mayouf et al., with appropriate modifications (Mayouf et al., 2019). To prepare the ABTS working solution, equal volumes of 7 mmol/L ABTS+ solution and 2.45 mmol/L potassium persulfate solution were mixed and left to stand for 16 h in the dark. Before use, the ABTS solution was diluted to an absorbance of approximately 0.7 at 734 nm. Subsequently, 100 μL of peptide samples were combined with 100 μL of diluted ABTS solution and incubated at 37 °C for 30 min in the dark. The absorbance was then measured at 734 nm. Deionized water and Vc served as negative and positive controls, respectively. The ABTS free radical scavenging rate was calculated using the following formula:

$$\mathrm{ABTS}+\mathrm{scavenging rate}\%=1-\frac{A_{2}}{A_{1}}\times 100\%$$

where A_1_ is the absorbance of the negative control and A_2_ is the absorbance of the peptide sample.

The hydroxyl radical scavenging capacity was based on the method described by Ma et al. with appropriate adjustments (Ma et al., 2020). To perform the assay, 100 μL of the sample solution, 35 μL of 3 % H2O2 solution, 50 μL of 5 mmol/L FeSO4 solution, and 15 μL of salicylic acid-ethanol solution were mixed. The absorbance was measured at 510 nm following incubation at 37 °C for 30 min. Deionized water and Vc were used as negative and positive controls, respectively. The hydroxyl radical scavenging rate was calculated using the following formula:

$$\mathrm{Hydroxyl radical scavenging rate}\%=\frac{1-A_{0}-A_{1}}{A_{2}}\times 100\%$$

Here, A0 represents the absorbance of the sample, A1 denotes the absorbance with absolute ethanol substituting for salicylic acid, and A2 represents the absorbance with absolute ethanol substituting for the sample.

### 2.15 Molecular docking analysis

The 3D structures of the small molecule compounds ABTS (CID: 9570474) and DPPH (CID: 2735032) were downloaded from the Pubchem database (https://pubchem.ncbi.nlm.nih.gov/). The 2D structures of LFW and LFPW were drawn using the ChemDraw software (Version 22.2.0) and converted to energy-minimized and stability-optimized 3D structures using Chem 3D. Molecular docking was performed using AutoDock Vina (Version 1.1.2)and the results were analyzed and visualized using PyMOL software (Trott & Olson, 2010).

### 2.16 Date analysis

All data represent the mean values of three replicate experiments and are presented as mean ± standard deviation. Statistical analyses, including ANOVA and Duncan's multiple range test, were performed using SPSS Statistics 22.0, with *p* < 0.05 considered statistically significant. Figures were generated using Origin 2018.

## 3 Results and discussion

### 3.1 Effect of different fermentation conditions on the body wall of *Apostichopus japonicus*

Table 3 demonstrates that as the bacterial inoculum volume and fermentation time increase, the sensory scores of AJBW fermentation broth initially rise and subsequently decline. The polypeptide and total free amino acid content gradually increase and then level off. When the inoculum volume is 3 %, the weighted composite score is significantly higher than those of other inoculum volume (*p* < 0.05). No significant difference exists in composite score between fermentation times of 36 h and 48 h, and both are significantly higher than other fermentation times (*p* < 0.05). This indicates that appropriate microbial fermentation can significantly degrade sea cucumber wall proteins into peptides and amino acids, yielding a pleasant fruity and refreshing sour taste. However, excessive inoculum or prolonged fermentation results in an overly strong fermentation flavor and sour taste, which deteriorate the overall sensory quality. At lower fermentation temperatures, the sensory scores of AJBW fermentation broth are higher with minimal variation. In contrast, at higher temperatures, the flavor and texture of fermentation broth rapidly decline. The polypeptide content and total free amino acid levels also first increase and then decrease. There is no significant difference in the weighted comprehensive scores between fermentation temperatures of 30 °C and 35 °C, both of which are significantly higher than other fermentation temperatures (*p* < 0.05). Therefore, the optimal fermentation conditions are a 3 % bacterial inoculum amount, 36 h of fermentation time, and a fermentation temperature of 30 °C.

**Table 3:** Effects of different fermentation conditions on the composite score of AJBW fermentation broth.

Factor	Level	Sensory score	Peptide content (mg/g)	Standardized peptide value (%)	Total free amino acid content (g/100 g)	Standardized free amino acid value (%)	Composite score	Significance marking
Inoculum volume (%)	0.5	60.02 ± 1.20	3.85 ± 0.29	60.63 ± 4.57	0.47 ± 0.03	50.00 ± 3.19	57.67 ± 1.52	d
	1	63.92 ± 0.69	5.26 ± 0.39	82.83 ± 6.14	0.69 ± 0.07	73.40 ± 7.45	71.02 ± 1.20	c
	3	74.32 ± 0.73	5.87 ± 0.26	92.44 ± 4.41	0.94 ± 0.05	100.00 ± 5.32	85.27 ± 1.05	a
	5	68.46 ± 1.03	6.26 ± 0.47	98.58 ± 7.51	0.89 ± 0.11	94.68 ± 11.70	82.55 ± 1.43	b
	7	58.94 ± 0.31	6.35 ± 0.73	100.00 ± 11.50	0.92 ± 0.09	97.87 ± 9.57	78.94 ± 1.89	c
Fermentation time (h)	12	46.90 ± 1.36	3.56 ± 0.42	56.06 ± 6.61	0.37 ± 0.03	39.36 ± 3.19	47.31 ± 1.67	d
	24	61.24 ± 0.94	4.84 ± 0.89	76.22 ± 17.52	0.60 ± 0.10	63.83 ± 10.64	65.63 ± 2.31	b
	36	78.74 ± 1.92	5.40 ± 0.24	85.04 ± 4.44	0.85 ± 0.08	90.43 ± 8.51	83.24 ± 1.07	a
	48	74.94 ± 0.98	5.76 ± 0.52	90.71 ± 9.03	0.82 ± 0.12	87.23 ± 12.77	81.96 ± 1.60	a
	60	52.04 ± 0.49	5.11 ± 0.94	80.47 ± 18.39	0.86 ± 0.12	91.49 ± 12.77	69.01 ± 2.09	c
Fermentation temperature (°C)	20	66.42 ± 0.79	3.60 ± 0.32	56.69 ± 5.04	0.45 ± 0.03	47.87 ± 3.19	59.35 ± 0.93	c
	25	68.92 ± 1.37	4.54 ± 0.34	71.50 ± 5.34	0.68 ± 0.07	72.34 ± 7.45	70.42 ± 1.39	b
	30	75.18 ± 2.19	5.79 ± 0.44	91.18 ± 7.60	0.74 ± 0.04	78.72 ± 4.26	80.07 ± 1.89	a
	35	72.54 ± 1.64	5.96 ± 0.23	93.86 ± 3.86	0.86 ± 0.09	91.49 ± 9.57	82.61 ± 1.35	a
	40	49.26 ± 1.04	5.21 ± 0.52	82.05 ± 9.98	0.80 ± 0.11	85.11 ± 11.70	66.42 ± 1.99	d

Notes: The independent variables were tested with a bacterial inoculum volume of 3 %, a fermentation time of 36 h, and a fermentation temperature of 30 °C as default values while each independent variable was changed. The data are shown as the mean ± standard deviation (n = 3). Different lowercase letters between groups of factors indicate significant differences (*p* < 0.05, Duncan's multiple range test, *n* = 3). Peptide and free amino acid contents were standardized using the maximum value method.

### 3.2 Effect of different enzymatic hydrolysis conditions on fermentation broth

Table 4 demonstrates that as the enzyme dosage increases, the sensory scores initially rise slightly and then continuously decline. The peptide content and total free amino acid levels gradually increase and then plateau. At an enzyme dosage of 500 U/g, the sensory scores are significantly higher than those at other dosages (*p* < 0.05). This indicates that an appropriate enzyme dosage can effectively degrade more proteins in the fermentation broth into peptides and free amino acids. However, an excessive enzyme dosage deteriorates the flavor and texture and intensifies specific tastes produced by enzymatic hydrolysis. As the enzymatic hydrolysis time increases, the sensory scores rise initially and then decline. The peptide content and total free amino acid levels initially increase and then plateau, peaking at 4 h of hydrolysis. This suggests that an appropriate hydrolysis time can further degrade proteins in the fermentation broth. However, prolonged hydrolysis deteriorates both flavor and texture. As the enzymatic hydrolysis temperature increases, the sensory scores, peptide content, and total free amino acid levels initially rise and then decline, peaking at 50 °C. Excessive hydrolysis temperature reduces protease activity and intensifies off-flavors resulting from hydrolysis. Therefore, the optimal single-factor enzymatic hydrolysis conditions are an enzyme dosage of 500 U/g, a hydrolysis time of 4 h, and a hydrolysis temperature of 50 °C.

**Table 4:** Effects of different enzymatic conditions on the composite score of AJM fermentation broth.

Factor	Level	Sensory score	Peptide content (mg/g)	Standardized peptide value (%)	Total free amino acid content (g/100 g)	Standardized free amino acid value (%)	Composite score	Significance marking
Emzyme dosage (U/g)	250	69.90 ± 1.26	7.36 ± 0.39	85.00 ± 4.50	0.98 ± 0.08	81.67 ± 6.67	76.62 ± 2.11	b
	500	76.06 ± 1.34	7.79 ± 0.47	90.00 ± 5.43	1.06 ± 0.10	88.33 ± 8.33	82.61 ± 1.82	a
	750	66.62 ± 1.09	8.14 ± 0.51	94.00 ± 5.90	1.05 ± 0.07	87.50 ± 5.83	78.69 ± 1.68	b
	1000	55.56 ± 1.12	8.56 ± 0.60	98.85 ± 6.93	1.14 ± 0.11	95.00 ± 9.17	76.24 ± 1.97	c
	2000	42.72 ± 1.11	8.66 ± 0.49	100.00 ± 5.66	1.12 ± 0.06	93.33 ± 5.00	69.69 ± 1.62	d
Enzymatic hydrolysis time (h)	2	68.24 ± 0.99	6.56 ± 0.37	75.75 ± 4.27	0.87 ± 0.03	72.50 ± 2.50	71.18 ± 1.24	c
	3	74.94 ± 1.64	6.92 ± 0.41	79.91 ± 4.74	1.07 ± 0.05	89.17 ± 4.17	79.74 ± 1.51	b
	4	78.74 ± 1.38	7.85 ± 0.48	90.65 ± 5.55	1.19 ± 0.12	99.17 ± 10.00	86.83 ± 1.78	a
	5	71.24 ± 0.95	8.36 ± 0.52	96.54 ± 6.01	1.14 ± 0.07	95.00 ± 5.83	83.51 ± 1.36	b
	6	52.04 ± 0.79	8.50 ± 0.61	98.15 ± 7.05	1.20 ± 0.06	100.00 ± 5.00	75.56 ± 1.63	d
Enzymatic hydrolysis temperature (°C)	30	73.04 ± 1.74	6.35 ± 0.49	73.33 ± 5.66	0.93 ± 0.04	77.50 ± 3.33	74.23 ± 1.81	b
	40	75.24 ± 1.46	7.04 ± 0.31	81.29 ± 3.58	0.97 ± 0.08	80.83 ± 6.67	78.15 ± 1.32	b
	50	79.20 ± 1.48	7.83 ± 0.46	90.41 ± 5.31	1.07 ± 0.06	89.17 ± 5.00	84.50 ± 1.43	a
	60	58.52 ± 1.13	6.88 ± 0.29	79.45 ± 3.35	1.15 ± 0.11	95.83 ± 9.17	73.08 ± 1.94	c
	70	54.12 ± 0.84	6.26 ± 0.48	72.29 ± 5.55	0.94 ± 0.09	78.33 ± 7.50	64.72 ± 1.56	d

Notes: The independent variables were tested with a complex protease dosage of 500 U/g, an enzymatic digestion time of 4 h, and an enzymatic digestion temperature of 50 °C as default values, while each independent variable was changed. The data are shown as the mean ± standard deviation (n = 3). Different lowercase letters between groups of factors indicate significant differences (*p* < 0.05, Duncan's multiple range test, *n* = 3). Peptide and free amino acid contents were standardized using the maximum value method.

### 3.3 RSM results

#### 3.3.1 Model equation building and significance test

Based on single-factor experimental results, enzyme dosage (A), bacterial inoculum volume (B), and fermentation time (C) were selected as independent variables, with the composite score as the dependent variable. The experimental conditions were established based on the design combinations provided by Design Expert 13 software, and the results are presented in Table 5.

**Table 5:** Experimental design and data for optimal conditions of co-fermentation of *Apostichopus japonicus* body wall using bacteria and emzyme.

	A (U/g)	B (%)	C (h)	Composite score
1	-1	-1	0	88.55
2	0	1	1	81.33
3	1	0	-1	87.87
4	0	0	0	92.38
5	0	0	0	93.12
6	0	1	-1	84.96
7	-1	0	-1	88.06
8	1	1	0	82.59
9	0	-1	-1	88.76
10	0	0	0	93.12
11	0	0	0	92.86
12	0	0	0	93.88
13	0	-1	1	83.95
14	1	0	1	81.89
15	-1	0	1	86.11
16	-1	1	0	84.58
17	1	-1	0	86.46

Analysis of the experimental data yielded the following second-order polynomial regression equation:$\;R=93.072-1.061A-1.858B-1.971C+0.025\mathrm{AB}-1.008\mathrm{AC}+0.445\mathrm{BC}-3.072A^{2}-4.455B^{2}-4.017C^{2}$. Variance and significance analyses were performed on the model, with the results presented in Table 6. The data indicate that the regression model is highly significant (*p* < 0.0001), with a non-significant lack-of-fit term (*P* = 0.9490 > 0.05), demonstrating a good fit and low experimental error. The regression model's coefficient of determination (R^2^), adjusted R^2^ (R^2^_Adj_), and coefficient of variation (CV) are 0.9955, 0.9898, and 0.4860 %, respectively. This indicates that over 97 % of the response value variation can be explained by this model, enabling accurate analysis and prediction of the experimental results.

**Table 6:** RSM regression model ANOVA.

Source	Sum of Squares	Degrees of Freedom (DF)	Variance	F Value	*p* Value
Model	285.60	9	31.73	173.55	< 0.0001
A	9.01	1	9.01	49.28	0.0002
B	27.60	1	27.60	150.96	< 0.0001
C	31.09	1	31.09	170.01	< 0.0001
AB	0.0025	1	0.0025	0.0137	0.9102
AC	4.06	1	4.06	22.21	0.0022
BC	0.7921	1	0.7921	4.33	0.0459
A^2^	39.74	1	39.74	217.35	< 0.0001
B^2^	83.56	1	83.56	456.97	< 0.0001
C^2^	67.95	1	67.95	371.62	< 0.0001
Residual	1.28	7	0.1829		
Lack of Fit	0.0987	3	0.0329	0.1114	0.9490
Pure Error	1.18	4	0.2953		
Cor Total	286.88	16			
R^2^	0.9955				
R^2^_Adj_	0.9898				

**Fig. 1:**
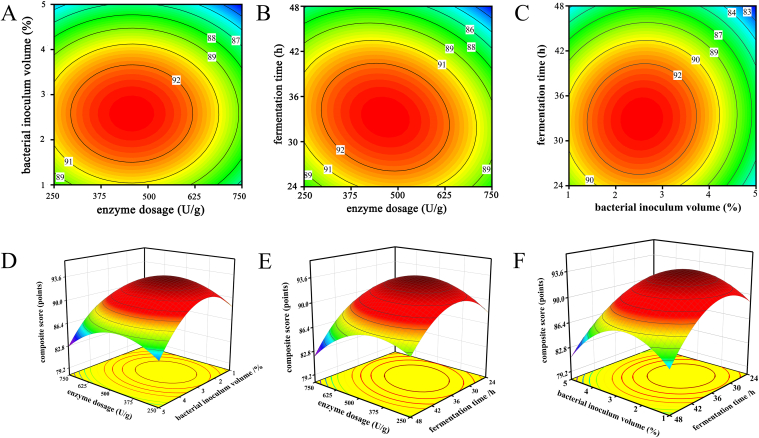
Contour plots and 3D response surface plots of the independent variables for the AJM composite score. (A) Contour plot of enzyme dosage and bacterial inoculum volume; (B) Contour plot of enzyme dosage and fermentation time; (C) Contour plot of bacterial inoculum volume and fermentation time; (D) 3D response surface plot of enzyme dosage and bacterial inoculum volume; (E) 3D corresponding surface plot of enzyme dosage and fermentation time; (F) 3D response surface plot of bacterial inoculum volume and fermentation time.

Fig. 1 and Table 6 show that the linear terms for enzyme dosage, bacterial inoculum volume, and fermentation time, as well as the quadratic terms for A^2^, B^2^, and C^2^, all have highly significant effects on the composite score (*p* < 0.01). Among the interaction terms, AB is not significant, while AC and BC significantly affect the composite score (*p* < 0.05). This indicates that enzyme dosage, bacterial inoculum volume, and fermentation time are significantly associated with the improvement of AJM flavor. Furthermore, based on the F-values, the primary factors influencing the overall flavor of AJM are fermentation time, followed by bacterial inoculum volume, and then enzyme dosage.

#### 3.3.2 Interactions between factors

We employed the multiple linear regression equation to create corresponding 3D surface plots and contour plots to illustrate the relationships between the response variable and different levels of treatment variables, as well as the interactions among these variables. The results demonstrate that enzyme dosage, bacterial inoculum volume, and fermentation time significantly affect the overall flavor of AJM, with complex interactions among these factors. Fig. 1A and D reveal that, with constant bacterial inoculum volume, the overall flavor of AJM initially increases and then decreases as the enzyme dosage increases. This indicates that a moderate enzyme dosage enhances the composite score of AJM, but an excessive enzyme dosage deteriorates the flavor, introducing a distinct enzymatic taste. Fig. 1B and E demonstrate that both excessive enzyme dosage and prolonged fermentation time result in a decrease in the composite score of AJM. Fig. 1C and F illustrate the relationship between bacterial inoculum volume and fermentation time. With constant bacterial inoculum volume, the composite score of AJM initially increases and then decreases with extended fermentation time. This may be due to substrate depletion, which limits bacterial growth, and the production of inhibitory by-products, thus diminishing the overall flavor of AJM.

#### 3.3.3 Co-fermentation process optimization and validation

The software-predicted optimal conditions were 463.50 U/g enzyme dosage, 2.34 % bacterial inoculum volume, and 31.06 h of fermentation, with a predicted composite score of 94.12. For practical purposes, the enzyme dosage was set at 463 U/g, bacterial inoculum volume at 2.3 %, and fermentation time at 31 h. Three parallel experiments were conducted according to the above conditions. The validation experiments yielded a composite score of 94.08 (closely matching the theoretical value), demonstrating the accuracy of the model predictions and the reliability of the optimization results.

Based on the optimized conditions, the total protein content in 50 g of AJBW homogenate was 2.27 ± 0.12 g. The final peptide content in AJM reached 8.05 ± 0.3 mg/g, and the total weight of the fermented product was 198.9 ± 0.14 g. Therefore, the total peptide yield was calculated as 1.6 ± 0.04 g , corresponding to a recovery rate of 70.48 ± 0.38 % from the initial protein content.

### 3.4 Detection of volatile flavor compounds

**Fig. 2:**
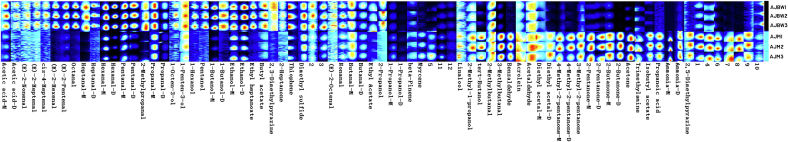
Gallery Plot of volatile flavor compounds in AJBW and AJM. Each bright spot in the plot represents a volatile compound, each row represents all signal peaks selected from one sample, and numerical numbers indicate substances that were not identified.

As shown in Table S1 and Fig. 2, GC-IMS was utilized to analyze the volatile flavor compounds in AJBW before and after BECF. A total of 65 volatile compounds were identified across both samples, including 14 alcohols, 26 aldehydes, 9 ketones, 3 acids, 4 esters, 1 ether, 2 hydrocarbons, and other compounds (ammonia, trimethylamine, 2,5-dimethylpyrazine, thiophene, 2,3-dimethylpyrazine). The most abundant compounds were aldehydes, alcohols, ketones, and hydrocarbons, which aligns with findings from other studies on the volatile flavor compounds in sea cucumber (Li, Dong, et al., 2022). Compared to untreated AJBW, the volatile flavor compounds in AJM exhibited significant differences in concentration. Notably, the concentration of low molecular weight aldehydes (e.g., hexanal, heptanal, octanal, and nonanal), which contribute to pungent or fishy odors, was significantly lower in AJM (Shao et al., 2023). This reduction in fishy odors is expected to have a substantial positive impact on the sensory qualities of AJM, as high levels of these aldehydes are commonly linked to undesirable flavors in seafood products. In contrast, the concentration of benzaldehyde, which has an almond-like aroma, was significantly higher (Hu et al., 2021). The increase in benzaldehyde is likely the primary contributor to the improved aroma profile of AJM, as it adds a sweet, pleasant note, thereby balancing the previously dominant fishy odor. The change in key flavor compounds and sensory impact in AJBW and AJM were presented in Table 7. Among alcohols, the contents of 1-butanol, pentanol, 1-hexanol, 1-penten-3-ol, and 1-octen-3-ol decreased or disappeared, while the levels of 1-propanol, linalool, and 2-methyl-1-propanol significantly increased. Pentanol, hexanol, and 1-octen-3-ol are typically considered products of lipid peroxidation (Li et al., 2020), whereas tert-butanol and linalool possess fresh, camphor-like aromas (Li, Hong, et al., 2022). In addition, BECF increased the levels of ketones, esters, and olefins in AJM. Ketones contribute significantly to the flavor profile despite their low odor threshold. For instance, acetone emits an aromatic odor, while 2-butanone and 2-pentanone emit odors similar to acetone, and 4-methyl-2-pentanone exhibits a pleasant ketone aroma (Ruan et al., 2021). In summary, BECF significantly enhanced the flavor profile of AJBW by reducing pungent aldehydes and increasing aromatic compounds such as benzaldehyde, ketones, esters, and olefins in AJM, thereby improving its overall flavor.

**Table 7:** Key flavor compounds variation and sensory impact.

Compound class	Compound	AJBW peak volune	AJM peak volume	Trend	Sensory contribution
Aldehyde	Benzaldehyde	221.80	895.34	↑404 %	Almond and sweet aroma
	Hexanal	3713.88	806.79	↓78 %	Reduced grassy, fishy odor
Alcohol	Ethanol	4581.83	2501.46	↓45 %	Reduced alcohol irritation
	tert-Butanol	193.30	1322.47	↑684 %	Fresh, camphor-like aromas
Acid	Acetic acid	1851.58	955.15	↓48 %	Less acidity and softer flavors
Ketone	2-Pentanone	310.92	1377.58	↑443 %	Noticeable fruity, sweet aroma
	4-Methyl-2-pentanone	30.67	186.41	↑608 %	Pleasant ketone-like odor

### 3.5 Determination of amino acid composition and content

To comprehensively assess the impact of BECF technology on the nutritional, flavor, and functional properties of AJBW, we employed an automated amino acid analyzer to determine the amino acid composition and content pre- and post- fermentation. As shown in Table 8, 17 amino acids were identified in both AJBW and AJM samples, with total amounts of 61.09 g/100 g and 58.10 g/100 g (dry weight), respectively. The essential amino acid totals were 19.25 g/100 g and 19.05 g/100 g (dry weight), respectively. The analysis revealed that key taste-active amino acids, including umami-enhancing glutamic acid (Glu) and aspartic acid (Asp), as well as sweet-tasting glycine (Gly), exhibited notably elevated concentrations in the fermented product. Notably, these amino acids primarily existed in peptide-bound forms, with free amino acids accounting for less than 1 % of total content. While free amino acids are recognized as direct contributors to taste perception, emerging evidence suggests that short peptides containing Glu and Asp residues may exert umami-enhancing effects through molecular synergism (Liu et al., 2019; Zhang et al., 2020). This phenomenon aligns with previous reports demonstrating that peptide-bound Glu exhibits distinct taste-modulating properties compared to its free form (Zhao et al., 2016). The observed amino acid profile underscores their dual functionality: Glu and Asp contribute to savory umami characteristics, while Gly enhances sweetness perception (Keska & Stadnik, 2017; Yu & Yang, 2020).

**Table 8:** The amino acid composition and content of AJBW and AJM (g/100 g, dry weight).

Amino acid	AJBW	AJM
Thr	3.12 ± 0.12	2.78 ± 0.31
Val	2.27 ± 0.06	2.33 ± 0.16
Met	0.87 ± 0.09	1.04 ± 0.04
Ile	1.73 ± 0.11	1.65 ± 0.08
Leu	2.62 ± 0.04	2.73 ± 0.07
Phe	1.44 ± 0.04	1.73 ± 0.12
Lys	1.87 ± 0.12	2.19 ± 0.27
His	0.60 ± 0.05	0.63 ± 0.15
Arg	4.73 ± 0.07	3.97 ± 0.25
Asp	6.03 ± 0.08	5.88 ± 0.22
Glu	9.80 ± 0.10	9.03 ± 0.36
Gly	10.31 ± 0.21	9.47 ± 0.54
Ser	3.07 ± 0.11	2.67 ± 0.22
Ala	4.38 ± 0.23	3.77 ± 0.36
Tyr	2.00 ± 0.13	2.29 ± 0.31
Cys	1.38 ± 0.15	1.46 ± 0.29
Pro	4.87 ± 0.09	4.48 ± 0.28

Notably, the AJM samples exhibited higher levels of methionine (Met), leucine (Leu), phenylalanine (Phe), lysine (Lys), and tyrosine (Tyr) compared to the AJBW samples. Research indicates that leucine (Leu) and lysine (Lys) are essential amino acids for human (Yuan et al., 2018), while tyrosine (Tyr) and phenylalanine (Phe) are critical for neurotransmitter synthesis (Vogt, 2010). Therefore, the increased levels of these amino acids suggest that BECF may enhance the nutritional value of the product.

However, overall, there is little difference in the amino acid composition of the two samples. This may be because, during microbial fermentation, microorganisms not only utilize nitrogen source nutrients to carry out their own metabolic activities, but also secrete proteases. These enzymes work synergistically with exogenously added enzymes to degrade the proteins in the body wall of sea cucumbers, resulting in more small molecule peptides and free amino acids. Due to this degradation process, the amino acids originally present in the sea cucumber body wall are released, thereby maintaining the overall amino acid composition. Therefore, although the specific content of each amino acid changes, the overall amino acid composition remains essentially the same.

### 3.6 Peptide sequence identification and its activity prediction

The *Apostichopus japonicus* peptide sample was analyzed using LC-MS/MS, and the total ion chromatogram obtained is shown in Fig. S1. The raw mass spectrometry data were processed using Byonic software, yielding 808 peptides with high confidence scores (>200). The sequence lengths ranged from 3 to 24 amino acids, with molecular weights ranging from 312.18 to 2824.30 Da.

Research indicates that some bioactive peptides have structural features that confer specific biological functions. PeptideRanker uses these structural characteristics to identify potentially bioactive peptides (Mooney et al., 2012). We screened the 808 identified peptides using the PeptideRanker database to predict and score their bioactivity. The top 10 peptides, listed in Table 9, had PeptideRanker scores above 0.95, suggesting potential bioactivity. Additionally, a search in the BIOPEP-UWM database revealed that these 10 peptides are novel. Safety assessments indicated that these peptides are non-toxic and non-allergenic. Other physicochemical properties are detailed in Table 9. Research indicates that short peptides (2–10 amino acids) often display greater biological activity compared to longer natural proteins (Ma et al., 2022). Zu et al. investigated the antioxidant activity of silver carp scale peptides with varying molecular weights through free radical scavenging assays and observed that low molecular weight peptides exhibited stronger antioxidant activity (Zu et al., 2022). Therefore, these high-scoring short peptides may have significant potential for future applications in biomedicine and functional food development.

**Table 9:** Bioinformatics analysis of the top 10 peptides in the PeptideRanker score.

Peptide	*Mr*	Theoretical *pI*	Hydrophilicity	Novelty of Peptide	Toxicity	Allergenicity	PeptideRanker Score
FDWF	613.71	3.80	−1.35	Yes	Non-toxic	Non-allergenic	0.996
LFW	464.60	5.88	−2.57	Yes	Non-toxic	Non-allergenic	0.995
GFDWF	670.78	3.80	−1.08	Yes	Non-toxic	Non-allergenic	0.993
LFPW	561.73	5.88	−1.92	Yes	Non-toxic	Non-allergenic	0.991
FIW	464.60	5.88	−2.57	Yes	Non-toxic	Non-allergenic	0.990
FNPF	523.63	5.88	−1.20	Yes	Non-toxic	Non-allergenic	0.988
IFPF	522.69	5.88	−1.70	Yes	Non-toxic	Non-allergenic	0.987
LGFF	482.63	5.88	−1.70	Yes	Non-toxic	Non-allergenic	0.986
LFGW	521.67	5.88	−1.92	Yes	Non-toxic	Non-allergenic	0.986
FGPSPF	650.80	5.88	−0.78	Yes	Non-toxic	Non-allergenic	0.976

### 3.7 Synthesis and in vitro antioxidant activity evaluation of identified peptides

**Fig. 3:**
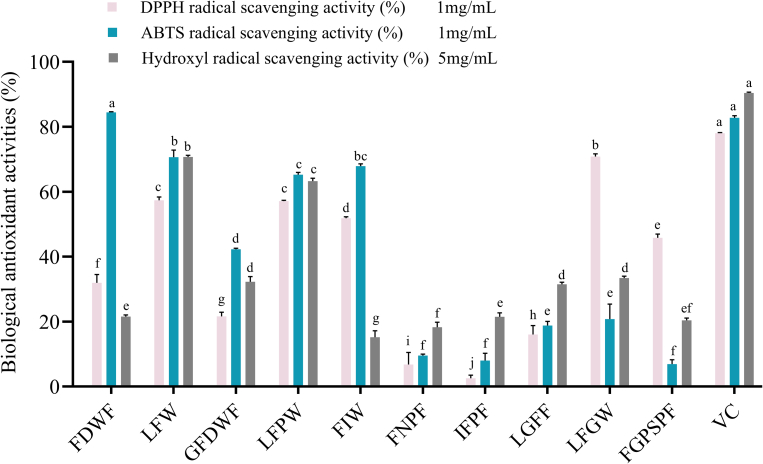
Total antioxidant activity of peptides with top 10 PeptideRanker scores. Data were expressed as means ± SD (*n* = 3), bar graphs with different lower letters are significantly different (*p* < 0.05).

To verify the antioxidant activity of these 10 peptides, we commissioned Shanghai Sangon Biotech Co., Ltd. to synthesize them and evaluated their abilities to scavenge DPPH, ABTS, and hydroxyl radicals. As shown in Fig. 3, all 10 peptides demonstrated varying levels of antioxidant activity, with LFW and LFPW showing particularly strong effects. At sample concentrations of 1 mg/mL, 1 mg/mL, and 5 mg/mL, the DPPH, ABTS, and hydroxyl radical scavenging rates for LFW were 57.36 ± 1.00 %, 70.61 ± 2.18 %, and 70.68 ± 0.51 %, respectively; the corresponding rates for LFPW were 57.11 ± 0.22 %, 65.23 ± 0.72 %, and 63.22 ± 0.91 %. The concentrations of 1 mg/mL and 5 mg/mL were selected in accordance with established methodologies for marine bioactive peptide characterization reported in prior studies (Yang et al., 2024). In comparison, at the same concentrations, the positive control, Vc, showed scavenging rates of 77.99 ± 0.21 %, 82.79 ± 0.62 %, and 90.41 ± 0.24 % for DPPH, ABTS, and hydroxyl radicals, respectively. The findings suggest that aromatic amino acids (especially F and W) and hydrophobic amino acids (such as L, F, and P) can enhance the activity of antioxidant peptides (Wang, et al., 2024). This aligns with our results, which may be due to the strong hydrogen-donating ability of these amino acids, making them effective radical scavengers (Zheng et al., 2016). However, further research is needed to elucidate their specific mechanisms of action.

### 3.8 Molecular docking analysis

**Fig. 4:**
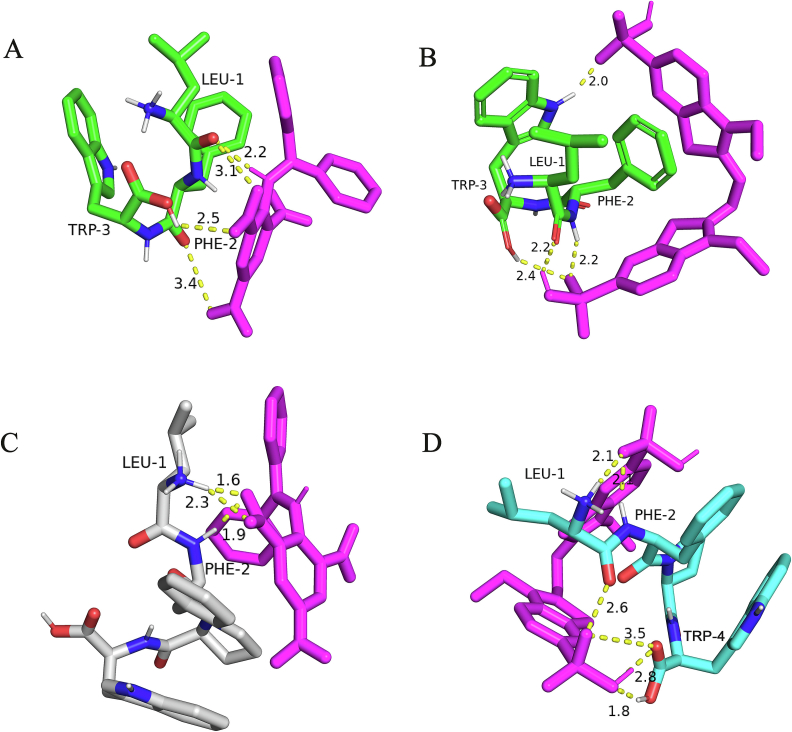
Molecular docking of LFW and LFPW with DPPH and ABTS radicals, respectively, and images obtained using PyMOL software. (A) LFW-DPPH; (B) LFW-ABTS; (C) LFPW-DPPH; (D) LFPW-ABTS.

Molecular docking is a widely used molecular modeling technique to predict interactions between proteins and small molecules, such as drugs or peptides (Chen et al., 2024). To better understand the antioxidant mechanisms of LFW and LFPW, we performed molecular docking analyses of these peptides with DPPH and ABTS free radicals using AutoDockTools 1.5.7 software. The results, illustrated in Fig. 4, show that the Leu and Trp residues in LFW formed four hydrogen bonds with the DPPH free radical, with a binding energy of −2.09 kcal/mol. Similarly, the Leu, Phe, and Trp residues in LFW formed four hydrogen bonds with the ABTS free radical, resulting in a binding energy of −3.94 kcal/mol (Fig. 4A and B). In contrast, the Leu and Phe residues in LFPW formed three hydrogen bonds with the DPPH free radical, with a binding energy of −4.41 kcal/mol. Meanwhile, the Leu, Phe, and Trp residues in LFPW formed six hydrogen bonds with the ABTS free radical, with a binding energy of −5.39 kcal/mol (Fig. 4C and D). Research indicates that lower binding energies typically correspond to stronger receptor-ligand interactions. For example, Chen Qianzi et al. studied the molecular docking of the antioxidant peptide WYR, derived from chicken liver by-products, with DPPH and ABTS free radicals. They found that WYR had a lower binding energy with ABTS (−3.04 kcal/mol) than with DPPH (−2.08 kcal/mol), suggesting a stronger radical-scavenging ability (Chen et al., 2024). Comparing the binding energies of LFW and LFPW with DPPH and ABTS, both peptides exhibited lower binding energies with ABTS than with DPPH, which aligns with previous free radical scavenging experimental results. Notably, although LFPW showed lower binding energies with both DPPH and ABTS compared to LFW, its radical-scavenging activity was slightly lower. This discrepancy may be due to the inhibitory effect of the Pro residue in LFPW on its antioxidant activity. In conclusion, both LFW and LFPW form multiple stable hydrogen bond interactions with DPPH and ABTS free radicals, indicating high antioxidant activity at the molecular level. These findings provide critical molecular insights into the mechanisms of these antioxidant peptides.

## 4 Conclusion

In this study, we aimed to optimize the process conditions of BECF for AJBW, to investigate the changes of the products during the fermentation process, and to screen the peptides with potential biological activities. Based on the results of the single-factor experiment, the BECF fermentation process was optimized using Design-Expert software. A mathematical model was established, determining the optimal conditions: 2.3 % bacterial inoculum, fermentation at 30 °C for 31 h; 463 U/g enzyme dosage, and enzymatic hydrolysis at 50 °C for 4 h. The optimized conditions were validated for reasonable and reliable. GC-IMS analysis demonstrated a significant reduction in volatile flavor compounds with pungent or fishy odors in AJM, while the concentrations of aromatic compounds such as alcohols, ketones, and esters were significantly increased. This indicates that BECF technology effectively reduces pungent odors. Amino acid analysis showed increased levels of essential amino acids in AJM. LC-MS/MS analysis identified 808 peptides, ranging in molecular weights from 312.18 to 2824.30 Da. Bioinformatics analysis indicated that the top 10 peptides, based on PeptideRanker scores, were novel, non-toxic, and non-allergenic. The antioxidant activities of these 10 peptides were further assessed using DPPH, ABTS, and hydroxyl radical scavenging assays, which revealed that LFW and LFPW demonstrated the highest antioxidant activities. Molecular docking analysis further confirmed that peptides LFW and LFPW formed multiple hydrogen bonds with DPPH and ABTS compounds. In summary, this study provides a promising approach to developing sea cucumber products with enhanced flavor and quality and provides a theoretical foundation for creating products with antioxidant and anti-aging properties.

## CRediT authorship contribution statement

**Zhiqiang Shu:** Writing – original draft, Validation, Software, Methodology, Investigation, Formal analysis, Data curation, Conceptualization. **Gongming Wang:** Writing – review & editing, Resources, Funding acquisition. **Yuexin Jing:** Supervision, Resources, Methodology. **Chunna Jiao:** Writing – review & editing, Supervision. **Leilei Sun:** Supervision, Investigation. **Hui Huang:** Supervision, Investigation. **Yue Li:** Validation, Data curation. **Jian Zhang:** Writing – review & editing, Supervision, Resources, Funding acquisition, Conceptualization.

## Declaration of competing interest

The authors declare that they have no known competing financial interests or personal relationships that could have appeared to influence the work reported in this paper.

## Data Availability

Data will be made available on request.

## Appendix A Supplementary data

Supplementary material 1 Image 1
