# Supplementary material for: Enhancement of Apostichopus japonicus peptide flavor through bacterial and enzyme co-fermentation (BECF) and the identification of novel antioxidant peptides in the fermented product
Source: Food Chem X. 2025 Feb 26;27:102323. doi: 10.1016/j.fochx.2025.102323 (PMC11999528; doi:10.1016/j.fochx.2025.102323)
Supplement: Supplementary file 1 — Supplementary material 1 [file mmc1.docx]

**^[[1]](#footnote-0)^Supplementary materials**

**Enhancement of *Apostichopus japonicus* peptide flavor through bacterial and enzyme co-fermentation (BECF) and the identification of novel antioxidant peptides in the fermented product**

Zhiqiang Shu ^a, b^, Gongming Wang ^a, c, ＊^, Yuexin Jing ^a, c^, Chunna Jiao ^a, c^, Leilei Sun ^d^, Hui Huang ^a, c^, Yue Li ^a, b^, Jian Zhang ^a, c, ＊^

^a^ Shandong Marine Resource and Environment Research Institute, Yantai 264006, PR China

^b^ Department of Food Science and Technology, Shanghai Ocean University, Shanghai 200120, PR China

^c^ Yantai Key Laboratory of Quality and Safety Control and Deep Processing of Marine Food, Yantai 264006, PR China

^d^ Yantai Key Laboratory of Characteristic Agricultural Bioresource Conservation & Germplasm Innovative Utilization, School of Life Sciences, Yantai University, Yantai 264005, PR China

**Table. S1** GC-IMS detection results

| **Class** | **Number** | **Compound** | **Molecular formula** | **Rt** | **Peak volume** | |
| --- | --- | --- | --- | --- | --- | --- |
|  |  |  |  |  | **AJBW** | **AJM** |
| Alcohol | 1 | Linalool | C_10_H_18_O | 1551.07 | 206.04 | 219.03 |
| Alcohol | 2 | 1-Octen-3-ol | C_8_H_16_O | 1231.16 | 165.19 | 120.75 |
| Alcohol | 3 | 1-Hexanol | C_6_H_14_O | 962.67 | 206.91 | 101.57 |
| Alcohol | 4 | Pentanol | C_5_H_12_O | 770.99 | 158.16 | 58.94 |
| Alcohol | 5 | 1-Butanol-M | C_4_H_10_O | 605.04 | 1206.13 | 298.58 |
| Alcohol | 6 | 1-Butanol-D | C_4_H_10_O | 605.04 | 63.28 | 15.07 |
| Alcohol | 7 | 1-Penten-3-ol | C_5_H_10_O | 635.02 | 471.52 | 716.42 |
| Alcohol | 8 | 2-Methyl-1-propanol | C_4_H_10_O | 507.29 | 91.50 | 100.23 |
| Alcohol | 9 | 1-Propanol-M | C_3_H_8_O | 430.11 | 376.40 | 527.18 |
| Alcohol | 10 | 1-Propanol-D | C_3_H_8_O | 431.02 | 56.86 | 77.45 |
| Alcohol | 11 | Ethanol-M | C_2_H_6_O | 324.37 | 4798.36 | 3772.71 |
| Alcohol | 12 | Ethanol-D | C_2_H_6_O | 324.78 | 4581.83 | 2501.46 |
| Alcohol | 13 | 2-Propanol | C_3_H_8_O | 315.76 | 59.50 | 11.97 |
| Alcohol | 14 | tert-Butanol | C_4_H_10_O | 310.02 | 193.30 | 1322.47 |
| Aldehyde | 15 | Pentanal-M | C_5_H_10_O | 367.85 | 2921.35 | 1938.90 |
| Aldehyde | 16 | Pentanal-D | C_5_H_10_O | 367.85 | 1842.91 | 549.79 |
| Aldehyde | 17 | 2-Methylbutanal | C_5_H_10_O | 313.71 | 1200.76 | 1036.04 |
| Aldehyde | 18 | 3-Methylbutanal | C_5_H_10_O | 313.71 | 705.05 | 5501.80 |
| Aldehyde | 19 | (E)-2-Nonenal | C_9_H_16_O | 1383.01 | 290.50 | 159.16 |
| Aldehyde | 20 | (E)-2-Octenal | C_8_H_14_O | 1116.31 | 123.27 | 83.23 |
| Aldehyde | 21 | Nonanal | C_9_H_18_O | 1037.16 | 390.46 | 290.44 |
| Aldehyde | 22 | Octanal | C_8_H_16_O | 827.29 | 629.96 | 129.25 |
| Aldehyde | 23 | (E)-2-Hexenal | C_6_H_10_O | 720.61 | 234.82 | 58.60 |
| Aldehyde | 24 | Heptanal-M | C_7_H_14_O | 673.19 | 529.60 | 58.43 |
| Aldehyde | 25 | Heptanal-D | C_7_H_14_O | 672.21 | 145.56 | 55.83 |
| Aldehyde | 26 | cis-4-Heptenal | C_7_H_12_O | 756.17 | 55.83 | 20.88 |
| Aldehyde | 27 | (E)-2-Heptenal | C_7_H_12_O | 883.59 | 76.15 | 41.56 |
| Aldehyde | 28 | (E)-2-Pentenal | C_5_H_8_O | 584.29 | 404.34 | 127.29 |
| Aldehyde | 29 | Hexanal-M | C_6_H_12_O | 494.63 | 4603.15 | 2148.64 |
| Aldehyde | 30 | Hexanal-D | C_6_H_12_O | 495.34 | 3713.88 | 806.79 |
| Aldehyde | 31 | Butanal-M | C_4_H_8_O | 287.05 | 443.90 | 364.52 |
| Aldehyde | 32 | Butanal-D | C_4_H_8_O | 287.46 | 96.30 | 79.55 |
| Aldehyde | 33 | Propanal-M | C_3_H_6_O | 245.63 | 2624.03 | 2008.65 |
| Aldehyde | 34 | Propanal-D | C_3_H_6_O | 246.04 | 1842.27 | 1033.54 |
| Aldehyde | 35 | 2-Methylpropanal | C_4_H_8_O | 253.83 | 192.40 | 35.61 |
| Aldehyde | 36 | Acrolein | C_3_H_4_O | 271.46 | 94.86 | 97.18 |
| Aldehyde | 37 | Acetaldehyde | C_2_H_4_O | 216.92 | 177.00 | 192.70 |
| Aldehyde | 38 | Diethyl acetal-M | C_6_H_14_O_2_ | 296.44 | 207.66 | 199.34 |
| Aldehyde | 39 | Diethyl acetal-D | C_6_H_14_O_2_ | 296.34 | 31.08 | 237.36 |
| Aldehyde | 40 | Benzaldehyde | C_7_H_6_O | 1421.21 | 221.80 | 895.34 |
| Aldehyde | 41 | 4-Methyl-2-pentanone-M | C_6_H_12_O | 410.97 | 203.00 | 993.73 |
| Ketone | 42 | 4-Methyl-2-pentanone-D | C_6_H_12_O | 409.48 | 30.67 | 186.41 |
| Ketone | 43 | 2-Pentanone-M | C_5_H_10_O | 366.70 | 310.92 | 1377.58 |
| Ketone | 44 | 2-Pentanone-D | C_5_H_10_O | 366.70 | 329.18 | 1389.78 |
| Ketone | 45 | 2-Heptanone | C_7_H_14_O | 657.63 | 42.28 | 19.58 |
| Ketone | 46 | 2-Butanone-M | C_4_H_8_O | 304.69 | 344.90 | 1263.10 |
| Ketone | 47 | 2-Butanone-D | C_4_H_8_O | 303.46 | 79.44 | 3799.89 |
| Ketone | 48 | Acetone | C_3_H_6_O | 255.47 | 1354.43 | 14363.85 |
| Ketone | 49 | 3-Methyl-2-pentanone | C_6_H_12_O | 393.93 | 91.53 | 209.32 |
| Acid | 50 | Propanoic acid | C_3_H_6_O_2_ | 1421.21 | 218.92 | 534.56 |
| Acid | 51 | Acetic acid-M | C_2_H_4_O_2_ | 1284.25 | 1851.58 | 955.15 |
| Acid | 52 | Acetic acid-D | C_2_H_4_O_2_ | 1284.25 | 1851.58 | 955.15 |
| Ester | 53 | Ethyl heptanoate | C_9_H_18_O_2_ | 902.14 | 456.71 | 167.07 |
| Ester | 54 | Butyl acetate | C_6_H_12_O_2_ | 474.07 | 105.15 | 54.74 |
| Ester | 55 | Ethyl Acetate | C_4_H_8_O_2_ | 292.79 | 252.17 | 62.33 |
| Ester | 56 | Isobutyl acetate | C_6_H_12_O_2_ | 402.03 | 65.27 | 276.83 |
| Ether | 57 | Dimethyl sulfide | C_2_H_6_S | 232.91 | 984.39 | 878.07 |
| Hydrocarbon | 58 | β-Pinene | C_10_H_16_ | 577.13 | 47.91 | 54.38 |
| Hydrocarbon | 59 | Myrcene | C_10_H_16_ | 630.57 | 32.77 | 90.92 |
| Other | 60 | Ammonia-M | H_3_N | 681.83 | 6806.39 | 221268.77 |
| Other | 61 | Ammonia-D | H_3_N | 683.29 | 21799.71 | 143330.02 |
| Other | 62 | 2,3-Dimethylpyrazine | C_6_H_8_N_2_ | 890.51 | 28.25 | 13.42 |
| Other | 63 | Thiophene | C_4_H_4_S | 401.04 | 2751.63 | 1301.94 |
| Other | 64 | Trimethylamine | C_3_H_9_N | 257.11 | 168.86 | 322.47 |
| Other | 65 | 2,5-Dimethylpyrazine | C_6_H_8_N_2_ | 874.76 | 32.93 | 76.45 |

Note: In the table, M represents monomer, D represents dimer, and Rt is retention time.


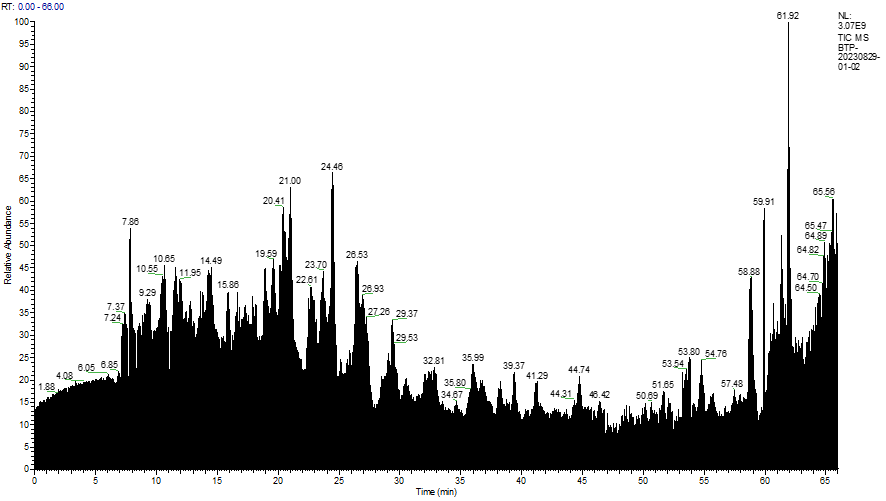


**Fig. S1.** Total ions chromatography of peptides

1. ＊Corresponding author.

   E-mail addresses: [s13970318519@163.com](mailto:s13970318519@163.com) (Z. Shu), [wgmsd105@163.com](mailto:wgmsd105@163.com) (G. Wang), [jyx2013xin@163.com](mailto:jyx2013xin@163.com) (Y. Jing), [jcnnn@126.com](mailto:jcnnn@126.com) (C. Jiao), [leilei.198966@163.com](mailto:leilei.198966@163.com) (L. Sun), [hh57319@126.com](mailto:hh57319@126.com) (H. Huang), [ly54329@163.com](mailto:ly54329@163.com) (Y. Li), [zjsd408@163.com](mailto:zjsd408@163.com) (J. Zhang). [↑](#footnote-ref-0)
